# Proton Pump Inhibitors Do Not Reduce the Risk of Esophageal Adenocarcinoma in Patients with Barrett’s Esophagus: A Systematic Review and Meta-Analysis

**DOI:** 10.1371/journal.pone.0169691

**Published:** 2017-01-10

**Authors:** Qiang Hu, Tian-Tian Sun, Jie Hong, Jing-Yuan Fang, Hua Xiong, Stephen J. Meltzer

**Affiliations:** 1 Division of Gastroenterology and Hepatology, Key Laboratory of Gastroenterology and Hepatology, Ministry of Health; State Key Laboratory for Oncogenes and Related Genes, Renji Hospital, School of Medicine, Shanghai Jiao Tong University, Shanghai Institute of Digestive Disease, Shanghai, China; 2 Division of Gastroenterology, Department of Medicine and Oncology, Johns Hopkins University School of Medicine, Baltimore, Maryland, United States of America; University of Pennsylvania, UNITED STATES

## Abstract

**Objectives:**

Proton pump inhibitors (PPIs) have been used for treatment of Barrett's esophagus (BE) for many years. However, the connection between PPIs and esophageal adenocarcinoma (EAC) in patients with BE has still been controversial. The current systematic review and meta-analysis was designed to evaluate the association between PPIs and the risk of EAC or high-grade dysplasia (HGD) in patients with BE.

**Methods:**

A systematic literature search of studies reporting the association between PPIs and the risk of EAC and/or HGD in patients with BE was conducted in PubMed, Embase, Web of Science and the Cochrane Library. Next, literature was screened using previously established criteria and relevant data were extracted from included studies. Finally, the software program Review Manage 5.2 was applied to aggregate data and analyze the results.

**Results:**

Nine observational studies, comprising five cohort and four case-control studies (including a total of 5712 patients with BE), were identified. Upon meta-analysis, PPIs were found to have no association with the risk of EAC and/or HGD in patients with BE (unadjusted OR 0.43, 95% CI 0.17–1.08). Analysis for duration response relationship revealed no significant trend toward protection against EAC or HGD with PPIs usage for >2~3 years (one study using 7-year cutoff) when compared to usage for shorter time periods (PPIs usage >2~3 years *vs*. <2~3 years: OR 0.91 (95% CI 0.25–3.31) *vs*. 0.91 (0.40–2.07)).There also was considerable heterogeneity between studies.

**Conclusion:**

No dysplasia- or cancer-protective effects of PPIs usage in patients with BE were identified by our analysis. Therefore, we conclude that clinicians who discuss the potential chemopreventive effects of PPIs with their patients, should be aware that such an effect, if exists, has not been proven with statistical significance.

## Introduction

Barrett’s esophagus (BE) is a condition in which the stratified squamous epithelium (SSE) of the distal esophagus under goes intestinal metaplasia (transformation to specialized columnar epithelium) [1]. Approximately 10% of patients with several longstanding chronic gastroesophageal reflux disease (GERD) will eventually develop BE as a complication of GERD [2]. In the general population, the prevalence of BE is estimated at 1–2%, with white males over 60 years of age predominantly affected [3]. As BE is the single most important risk factor for the development of esophageal adenocarcinoma (EAC), and since the incidence of EAC has increased exponentially over the past 3 decades, increased attention has been focused on preventing the progression from BE to EAC or its immediate precursor lesion, high-grade dysplasia (HGD) [4–7].

Proton pump inhibitors (PPIs) are the most commonly prescribed class of medications that are used for treating GERD. PPIs are quite effective and remarkably safe [8]. There have been limited studies and debate regarding the effect of such treatment on the risk of progression to dysplasia cancer. On the one hand, reduction of esophageal acid exposure by PPIs decreases inflammation and proliferation. Additionally, proposed beneficial effects of PPIs include anti-oxidant properties [9], effects on neutrophils, endothelial cells, epithelial cells [10], and anti-apoptotic cell modulation [11]. Moreover, PPIs are thought to inhibit binding to adhesion molecules in malignant cells and to suppress metastasis[12].On the other hand, PPIs therapy interferes with esophageal exposure to secondary bile acids, and increases circulating gastrin levels, which may induce proliferation, COX-2 upregulation, and perhaps expansion of metaplasia. It has been suggested that PPIs may promote the development of Barrett’s metaplasia and its progression to dysplasia or cancer [13]. Epidemiological studies of the association between PPIs and EAC risk have also yielded conflicting conclusions. Some studies have suggested that PPIs exert a protective effect against progression from BE to EAC. Singh *et al* carried out a previous meta-analysis whose results also suggested a protective effect of PPIs [14]. However, since an accumulating number of increasingly inconsistent results have now been reported, we felt the need to again carefully analyze existing data to formulate an objective overview of this topic.

## Methods

### Study identification

To identify relevant studies, we used the Preferred Reporting Items for Systematic reviews and Meta-Analyses for Protocols 2009 (PRISMA- 2009) for this systematic review and meta-analysis (S1 Table). We conducted a systematic literature search of Medline, Embase, Web of Science and the Cochrane Library, with retrieval until January 2016. By combining the use of medical subject heading terms and keywords, including ‘proton pump inhibitor*’, ‘PPI’, ‘acid suppress*’, ‘omeprazole’,‘pantoprazole’, ‘esomeprazole’, ‘lansoprazole’, ‘rabeprazole’, ‘dexlansoprazole’ AND ‘barrett’s’ OR‘oesophageal’ AND ‘neoplasia’, ‘high-grade dysplasia’, ‘oesophageal adenocarcinoma’, we searched randomized controlled trials (RCTs) or observational studies (cohort and case–control design) in the above databases. This computer search was also supplemented by manual searches of the reference lists of all retrieved studies, review articles and conference abstracts. Following these searches, based on specified inclusion criteria (research object being patients with BE, study compared differences in incidence of HGD or EAC after taking PPIs *vs*. not) and exclusion criteria (non-observational studies, studies without knowledge of BO status, studies without sufficient information on progression to OAC or BO-HGD, and studies comparing medical and surgical therapy for GERD and BO), two authors (Q.H and TT.S) independently screened the retrieved documents by browsing the title and abstract. Inclusion was not otherwise restricted by study size, language or publication type. We excluded letters to the editor, review articles, case reports and animal experimental studies. When multiple reports describing the same population cohort were published, the most recent or complete report was used. Differences were resolved by discussion or consultation with a third researcher (H.X) as needed. Since our study was a review of previous published studies, ethical approval or patient consent was not required.

### Data extraction and quality assessment

After study identification, data from the included studies were extracted and summarized independently by two of the authors (Q.H and TT.S). We collected clinical information including patient characteristics, time and dosage of PPIs, potential confounding variables, and estimates of association. By using non-PPI users as a reference, we measured the association between patients exposed to PPIs for a specified time (<2~3 years or >2~3 years) *vs*. non-use to estimate duration–response relationship. Any disagreement was resolved by senior authors (H.X). We used the Newcastle–Ottawa scale to evaluate the methodological quality of case–control and cohort studies [15]. The Newcastle-Ottawa scale consists of three factors: patient selection, comparability of study groups, and assessment of outcome. A score of 0–9 (allocated as stars) was allocated to each study. Due to the theme of some included studies is not at all the same with this meta-analysis, we combine NOS and the level of theme similarity in these studies with this meta-analysis for study quality assessment. Any discrepancies were addressed by a joint re-evaluation of the original article.

### Outcomes assessed

Not only did we assess the risk of progression to EAC and/or HGD in patients with BE between PPIs users and non-users, but we also analyzed effects of time and dosage of PPIs on progression to EAC and/or HGD. Based on the time of PPI usage (>2~3 years *vs*. <2~3 years), study design (cohort *vs*. case–control), number of outcomes (>60*vs*. <60) and mean follow-up time (>5years *vs*. <5years or not recorded), we performed pre-planned subgroup analysis. To assess the presence of a reflux-independent association between PPI usage and risk of progression to EAC and/or HGD, we performed an analysis restricted to studies which adjusted for the presence of erosive esophagitis or reflux symptoms; Similarly, we restricted our analysis to studies which adjusted for concomitant use of non-steroidal anti-inflammatory drugs (NSAIDs)/aspirin or statins to assess the presence of any independent chemopreventive association. Due to time-related biases have affected several observational studies reporting impressive results on the effectiveness of certain medications in reducing the incidence of major disease outcomes, we made a detailed analysis regarding the risk of time-related biases in the individual studies according to the methods provided by literatures [16, 17].

### Statistical analysis

All analyses were performed using Review Manager 5.2(Cochrane Collaboration, Oxford, UK). Because two studies contributing to the estimate reported only the odds ratio (OR) and their 95% confidence intervals (CIs) [18, 19], we used the generic inverse variance method to include data in this meta-analysis. Since outcomes (*i*.*e*., progression to HGD/EAC) were relatively rare, Odds ratios (ORs) were considered approximations of RRs or HRs, and ORs were used to compare dichotomous variables. All results were reported with 95% CI. Statistical heterogeneity between studies was assessed using the chi-square test with significance set at *p* < 0.10, and heterogeneity was quantified using the I^2^ statistic. The random-effects model was used if the value of I^2^ was >70% between studies; otherwise, the fixed-effects model was used. Given the small number of studies identified in our analysis, statistical tests for assessing publication bias were not performed [20].

## Results

### Study selection and characteristics

**Fig 1:**
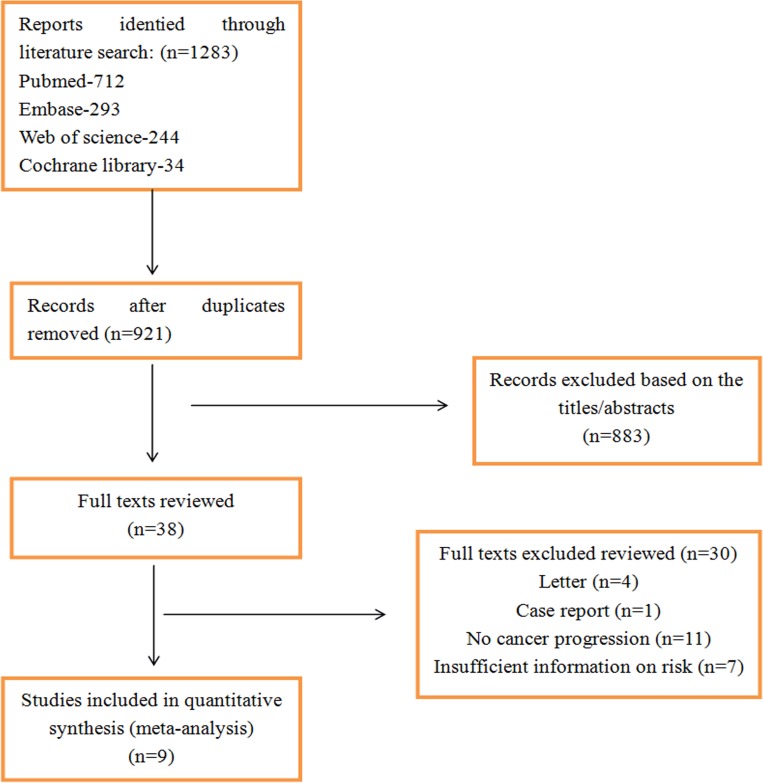
Flow diagram showing results of search and reasons for exclusion of studies.

In our initial search, we identified 1,283 article records in databases. After removing duplicate documents, 921 articles were identified. We browsed titles and abstracts to further exclude irrelevant literature. After this browsing, there were 38 potentially eligible studies assessed for inclusion. Finally, nine studies (five cohort [18, 19, 21–23] and four case-control [24–27] studies) satisfied our stringent inclusion criteria. The study flow is shown in Fig 1. The principal characteristics of the 9 included studies are presented in Table 1. Among them, four were conducted in the USA, three in the Netherlands, one in Australia, one in the UK, and one in Denmark. Sample sizes ranged from 77 to 1437(total 5712), of whom 501 progressed to EAC and/or HGD. The mean age of patients at the time of BE diagnosis ranged from 58 to 65 years, and approximately 73.1% of patients with BE were men. Only three studies interpreted study population race [21, 24, 25]. In addition to the use of PPIs, a proportion of patients also used non-steroidal anti-inflammatory drugs (NSAIDs)/aspirin and statins.

**Table 1:** Characteristics of individual studies included in the search.

Study	Location	Time period; follow up	Age at BE diagnosis	Sex (%men)	Race (%Caucasian)	Obesity (%with BMI>30kg/m^2^)	Smoking (% smokers)	Length of BE (% with LSBE)
Cohort studies								
Hillman.2004	Canberra,Australia;	1981–2001; median4.7years	58 (12)	71	NR	NR	NR	45.0
Nguyen.2009	Arizona, USA;	1982–2004; mean 7.6years	61 (12)	94	90	NR	NR	29.1
Altawil.2011	Michigan, USA;	2004–2010; NR	60	96	75	28.9	NR	NR
Jung.2011	Minnesota, USA;	1976–2006; median 5.9years	63 (14)	69	NR	29	13 (current)	59
Kastelein .2013	Rotterdam, The Netherlans;	2003–2009; mean 5.2years	61 (53–68)	71	NR	19	19 (current)	100
Case-control studies								
de Jonge .2006	Rotterdam, The Netherlans;	2003–2005; NA	62 (11)	74	100	27	20	NR
Nguyen .2010	Nationwide VA,USA;	2000–2002; NA	65 (10)	97	74	NR	NR	NR
Hivd-Jensen .2014	Nationwide Denmark	1995to2009 median 10.2 years	62.6 52.4–72.9	66.5	NR	NR	NR	NR
Masclee.2015	UK	1996to2011	64.8 (SD13.8)	63	NR	18.4	11.4 (current)	NR
	The Netherlans	1996to2012	61.2 (SD13.4)	62	NR	11.4	49.5 (current)	NR

### Quality assessment

Due to the fact that our included studies were either cohort or case-control, we applied the Newcastle-Ottawa scale to assess their methodological quality. In overall quality score (maximum = 9), according to the scores of specific tables, the methodological quality of the included studies was moderate to high, ranging from 6 to 9. After combining with the level of theme similarity in these studies with this meta-analysis, we divided these included studies into high quality [21, 22, 25–27] and moderate quality [18, 19, 23, 24]. Only four studies assessed obesity in BE patients [19, 22, 24, 26]. Three studies accounted for reflux symptoms [19, 22, 24] and four for erosive esophagitis [19, 22, 24, 27].

### PPI use and the risk of advanced neoplasia

**Fig 2:**
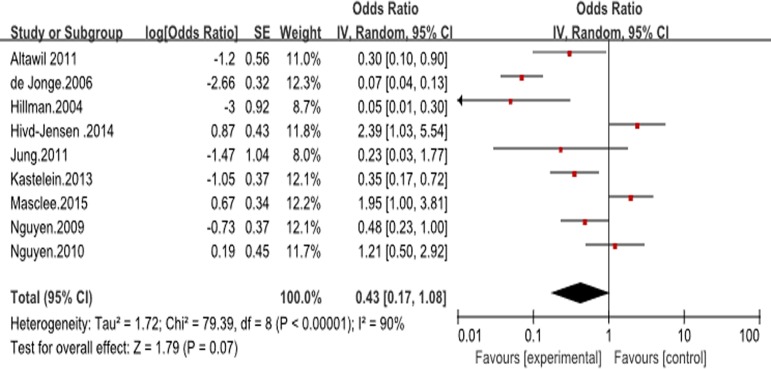
Forest plot of assessing the effects of PPIs on the patients with Barrett’s esophagus (BE) and the risk of esophageal adenocarcinoma (EAC) and/or high-grade dysplasia (HGD) in all included studies.

With the incidence of EAC and/or HGD as an endpoint, no significant difference was identified between PPI users and non-users (unadjusted OR 0.43, 95% CI 0.17–1.08) (Fig 2). In six studies, which reported the time to progression to EAC or HGD in a cohort of patients with BE, PPI users were also not significantly different from nonusers (HR 0.61, 95% CI 0.28–1.34) [17, 19, 21, 22, 27, 28]. Only three studies assessed the association of PPI dosage with the risk of progression to EAC and/or HGD (defined daily dose [DDD] as evaluation reference), however, all 3 of these studies showed no statistical significance [22, 26, 27]. There was insufficient information in these studies to allow estimation of PPIs’ effect on the risk of progression to EAC alone or to HGD alone. Considerable heterogeneity was observed in the overall analysis (I^2^ = 90%), although this was primarily due to the different sample sizes: when the sample size was less than 600, the use of PPIs was strongly associated with a lower risk of dysplasia in patients with BE (OR 0.20, 95% CI 0.09–0.46; I^2^ = 77%) [18, 19, 21–24]. In contrast, three studies in which the sample sizes were more than 600, showed an inconsistent result (OR 1.83, 95% CI 1.16–2.86; I^2^ = 0%)[25, 27, 28]. The use of PPIs and other medication and the incidence of EAC and/or HGD in included studies are particularly showed in Table 2.

**Table 2:** The use of PPIs and other medication and the incidence of EAC and/or HGD in included studies.

Study	Total no. Of patients with BE with baseline dysplasia status	Incident EAC and/or HGD	Patients on PPI		Patients not on PPI		Reflux symptoms; endoscopic esophagitis	Other medication use	
			Incident EAC and/or HGD	Total no. Of patients on PPI	Incident EAC and/or HGD	Total no. Of patients not on PPI		NSAIDs/aspirin	Statins
Cohort studies									
Hillman.2004	350 NDBE—85.4% LGD—14.6%	HGD—9 EAC—7 Combined—11	NR	NR	NR	NR	NR; 88%	78 (22.0%)	NR
Nguyen.2009	344 NDBE—100% LGD—0	HGD—20 EAC—13 Combined—33	17	231 (67.2%)	16	113 (32.8%)	NR	169 (49.1%)	87 (25.3%)
Altawil.2011	77 NDBO—100% LGD—0	17	7	49	10	28	NR	20 (26.0%)	27 (35.1%)
Jung.2011	355 NDBE—83% LGD—17%	HGD—12 EAC—7 Combined—19	NR	NR	NR	NR	77%; 31%	NR	NR
Kastelein.2013	540 NDB—86% LGD—14%	HGD—28 EAC—12 Combined—40	28	462 (85.6%)	12	78 (14.4%)	29%; 9%	110 (20.4%)	102 (18.9%)
Case-control studies									
de Jonge.2006	335	EAC—91	43	270 (81.6%)	44	61 (18.4%)	72.5%; NR	134 (40.0%)	NR
Nguyen.2010	812	EAC—116	110	763 (94.0%)	6	49 (6.0%)	NR	468 (57.6%)	377 (46.4%)
Hivd-Jensen .2014	1437 NDBE—89.8% LGD—10.2%	HGD—80 EAC—60 Combined—140	134	1306	6	131	NR	966(67.2%);439(30.5%)	250 (17.4%)
Masclee.2015	1466	57	46	1005	11	461	NR;4%	128(22.8%);183(26.3%)	248 (35.6%)
							NR;30%	104(13.5%);48(6.2%)	126 (16.4%)

### Subgroup and sensitivity analysis

When exposure time intervals were dichotomized as either short term (<2~3 years) or long term (>2~3 years), the association between PPI usage and risk of EAC or HGD was not statistically significant (Table 3). When classified by study design, case-control studies showed a result (OR 0.78, 95% CI 0.13–4.7) similar to all studies, however, cohort studies, though accounting for 29% of the total sample size, suggested a protective association between PPIs and the risk of EAC and/or HGD (OR 0.31, 95% CI 0.18–0.54). Since most of patients in the entire BE cohort were taking PPIs, statistically significant risk estimates were not noted. Regarding assessment of time-related biases, we had not found any of them in these four studies [22, 25–27]. One study existed immortal time bias, but we could not use a proper person-time approach to eliminate this bias from its provided data [21]. The data from other four studies were such unspecific that we could not evaluate whether or not they existed time-related biases [18, 19, 23, 24]. We also systematically excluded each study from the main summary estimate to assess whether any single study had a dominant effect on the summary OR. Result revealed that no single study markedly affected the summary estimate or *p* value for heterogeneity among the other summary estimates, and the pooled point estimate remained statistically insignificant (range 0.16–1.51), with the corresponding 95% CI bounds including 1.

**Table 3:** Subgroup analyses and duration–response relationship on the association of PPIs use and risk of EAC and/or HGD in patients with BE.

Groups	Categories	No. of studies	Adjusted OR	95% CI	Heterogeneity within groups (I^2^)	P _interaction_
Study design	Cohort	5	0.31	[0.18, 0.54]	26	0.34
	Case–control	4	0.78	[0.13, 4.70]	96	
Number of outcomes	<60	6	0.39	[0.16, 0.95]	79	0.07
	>60	3	0.58	[0.06, 5.81]	96	
mean follow-up time	<5years or no record	5	0.20	[0.04, 0.94]	90	0.13
	>5years	4	0.80	[0.33, 1.94]	81	
Duration–response	<2~3 years	5	0.91	[0.40, 2.07]	80	1.00
	>2~3years	5	0.91	[0.25, 3.31]	92	
Study quality	High	5	0.98	[0.46, 2.10]	80	<0.001
	Moderate	4	0.12	[0.05, 0.29]	53	
Time related bias	No	4	1.18	[0.49, 2.85]	81	0.005
	Yes or unclear	5	0.17	[0.06, 0.48]	78	

## Discussion

This systematic review and meta-analysis of nine observational studies was performed to evaluate the effect of PPIs on progressing to HGD and/or EAC in patients with BE. In this meta-analysis, published studies included a total of 5712 patients with non-dysplastic BE (or LGD), of whom 501 progressed to EAC and/or HGD. Our results conflict with results of previous studies, most of which reported an inverse relationship between PPI use and the risk of neoplastic progression, as well as a decreased risk of neoplastic progression with prolonged PPI use. A previous meta-analysis on this topic was performed by Singh *et al* and published in 2014[14]. This previous article included four cohort and two case-control studies for analysis, as partly described here, involving a total of 2813 patients, and showed that PPI use was associated with a 71% risk reduction in progression to EAC and/or HGD in a duration-dependent manner. We included two new studies (NOS = 7, 8) [26, 27]. Although these new included studies were case-control, they presented several advantages in study quality. Firstly, they featured a large cohort, including BE patients nationwide, the use of registries with validated high data coverage, and a complete prescription and hospitalization history. Secondly, nested case–control design in a well-defined population represents the general population minimized selection bias. In addition, because all prescription medications were recorded prospectively, there was no recall bias, and the use of the unique civil registration numbers allowed population-based design, complete follow-up, and linkage across registries. But most of all, both the two newer studies found no evidence of a protective effect from PPIs on the development of OAC or HGD, which results were in contrary with the conclusion of the previous meta-analysis. Hvid-Jensen *et al* identified the RR of OAC or HGD was 2.2 (95% CI: 0.7–6.7) and 3.4 (95% CI: 1.1–10.5) in long-term low- and high-adherence PPI users respectively. Masclee *et al* found PPIs used at highest dose showed an OR for HGD–OAC of 0.9 (95% CI 0.3 to 2.3). Therefore, because of these conflicting results, we seriously considered the relationship between these two studies and the previous meta-analysis.

When considering PPIs as potential chemopreventive agents, the protective mechanism likely involves decreasing intra-esophageal acid and bile exposure, thus promoting esophageal mucosal healing. The up-regulated production of cyclooxygenase-2 (COX-2) has been implicated in the progression of BE to EAC [29]. Because acid and bile exposure have been shown to increase COX-2 expression, PPIs should in theory counter this effect. Indeed, in experimental studies, COX-2 inhibitors suppressed the growth of BE cells, potentially through suppression of basic fibroblast growth factor [30]. Another study confirmed that prostaglandin E2, the product of COX-2 conversion, is reduced in patients with BE taking esomeprazole combined with high doses (325mg/day) of aspirin [31]. It is likely that a large proportion of registered PPI usage is symptom-driven, and reflux symptoms have also been associated with the risk of EAC even in persons without known BE [32]. The observation that PPIs may increase the risk of EAC is explained by the treatment indication being a risk factor for EAC, *i*.*e*., reverse causation and the phenomenon of ‘channeling’, where in high-risk patients are being prescribed high-dose PPIs whereas low-risk patients are being prescribed lower doses or not at all [25, 33, 34]. Therefore, it may be the severity of reflux that predisposes to cancer, rather than the usage of PPIs *per se*. However, in contravention with this hypothesis, the increased use of PPIs (introduced in the late 1980s [35]), is associated with the increasing incidence of EAC [36, 37]. Concerns that PPI-induced hypergastrinaemia may increase the risk of adenocarcinoma development have also been expressed [38]. *In vitro* studies have revealed that gastrin has a pro-proliferative effect on Barrett’s epithelium [39]. A potential causal effect of gastrin on neoplastic progression in human BE has been supported by one study showing that serum gastrin levels were significantly correlated with cellular proliferation in nondysplastic BE patients on PPI therapy [40]. Moreover, it is well-known that reflux symptoms correlate poorly with the actual amount of refluxate in patients with GERD, and that BE may even make patients hyposensitive to acid refluxate [41]. PPI usage and severity of reflux are therefore not necessarily linearly corrected. Hence, the risk correlation between PPIs and incidence of EAC reflects the therapeutic picture–not measurable reflux [42, 43]. This is in line with national guidelines, which recommend PPIs for symptom control alone, and not for the prevention of EAC [44, 45].

Our analysis also has several limitations that must be taken into account when interpreting our results. Firstly, our meta-analysis included only observational studies, which lacked the experimental random allocation of intervention necessary to test exposure–outcome hypotheses optimally. No RCTs have been performed to explore this association. We also did not have complete information about body mass index, tobacco and alcohol consumption, or H. pylori status, which may be important factors in neoplastic progression. Thirdly, several studies lacked detailed pathologic information on Barrett’ s segment length and grade of dysplasia, as is current practice for risk stratification of patients with BE. This deficiency may have resulted in misclassification of BE and EAC. In addition, we were unable to rule out publication bias. With such a limited number of studies, statistical testing for publication bias assessment is not recommended.

## Conclusions

In summary, no definitive protective effects against the development of EAC and/or HGD were seen for patients with BE with long-term PPI usage. Until and unless results of future studies can confirm such an association, PPI usage should be restricted to symptom control according to current guidelines. These findings indicate that for an unselected group of patients with BE, chemoprevention by use of PPIs to reduce progression should not be considered directly as routine care.

## Study Highlights

### What is current knowledge

The connection between PPIs and esophageal adenocarcinoma (EAC) in patients with Barrett's esophagus (BE) has still been controversial.

### What is new here

No definitive protective effects against the development of EAC and/or HGD were seen for patients with BE with long-term PPI usage.

PPIs were found to have no association with the risk of EAC and/or HGD in patients with BE (unadjusted OR 0.43, 95% CI 0.17–1.08).

## Supporting Information

S1 Table Protocol for this systematic review and meta-analysis using PRISMA-2009. (DOC)

## Abbreviations

PPIs: Proton pump inhibitors
BE: Barrett's esophagus
EAC: esophageal adenocarcinoma
HGD: high-grade dysplasia
SSE: stratified squamous epithelium
GERD: gastroesophageal reflux disease
